# A Rare Case of Levetiracetam-Induced Hemolytic Anemia

**DOI:** 10.7759/cureus.105064

**Published:** 2026-03-11

**Authors:** Pedro D Gil de Rubio Cruz, Dorimar Morales Torres, Karolane A Gonzalez Gonzalez

**Affiliations:** 1 Department of Internal Medicine, University of Puerto Rico at Río Piedras, San Juan, PRI; 2 Department of Internal Medicine, University of Puerto Rico Hospital - Dr. Federico Trilla, Carolina, PRI; 3 Health Services Administration, Centro Comprensivo de Cáncer de la Universidad de Puerto Rico, San Juan, PRI; 4 Department of Medicine, Universidad Central del Caribe, Bayamón, PRI

**Keywords:** alzheimer's disease and epilepsy, anti-seizure medication, drug induced adverse effect, geriatric pharmacology, levetiracetam-induced hemolytic anemia

## Abstract

This is the case of a 77-year-old female patient with Alzheimer’s disease, complicated by partial complex seizures. Levetiracetam was administered, which provoked hemolytic anemia. An extensive laboratory evaluation was suggestive of a non-immune etiology. Subsequent discontinuation of levetiracetam resulted in improvement of hemoglobin. Pancytopenia has been reported with the use of levetiracetam, but not isolated anemia. With this case report, we want to increase awareness of a potentially serious adverse reaction provoked by levetiracetam, a preferred anticonvulsive drug in the geriatric population.

## Introduction

Epilepsy is a common comorbidity in patients with Alzheimer’s disease and is associated with a poor course of cognitive symptoms, including accelerated cognitive decline and increased morbidity [1]. The incidence of seizures in patients with Alzheimer’s disease is significantly higher than in age-matched controls [2], and seizure disorders in this population have been associated with worse clinical outcomes and increased healthcare utilization [3]. Subclinical and focal seizures, in particular, may present with atypical neuropsychiatric manifestations such as confusion, agitation, or episodic behavioral changes, making diagnosis challenging in older adults with underlying neurodegenerative disease [3].

Levetiracetam (LEV) is commonly selected as anticonvulsive therapy in the geriatric population due to its effectiveness and safety profile [3]. LEV is frequently used as first-line therapy because of its broad efficacy, favorable pharmacokinetic properties, minimal drug-drug interactions, and general tolerability in older adults. Unlike many older antiepileptic drugs, LEV is not extensively metabolized by the liver and does not significantly induce or inhibit cytochrome P450 enzymes, making it particularly appealing in elderly patients with multiple comorbidities and polypharmacy [4]. As a result, LEV is commonly prescribed for focal and generalized seizures in patients with cognitive impairment and dementia [3].

Despite its overall favorable safety profile, LEV has been associated with a range of adverse effects, most commonly neuropsychiatric symptoms such as irritability, agitation, and mood changes [4]. Hematologic adverse reactions are considered rare, though cases of leukopenia, thrombocytopenia, and pancytopenia have been reported. However, isolated hemolytic anemia related to LEV use has not been well described in the literature.

Here, we present a rare case of LEV-induced hemolytic anemia in an elderly patient with suspected Alzheimer’s disease. This case highlights an uncommon but potentially serious adverse reaction to a widely used anticonvulsant and underscores the importance of vigilance and hematologic monitoring when initiating LEV in older adults, particularly those with complex neurologic and autoimmune comorbidities.

## Case presentation

A 77-year-old Hispanic female patient with hypertension and Sjögren syndrome had multiple episodes of disorientation, forgetfulness, wandering around, and agitation for two years. These symptoms led to several visits to the emergency department, some against her will. A major neurocognitive disorder was suspected, but further evaluation did not occur. 

**Figure 1:**
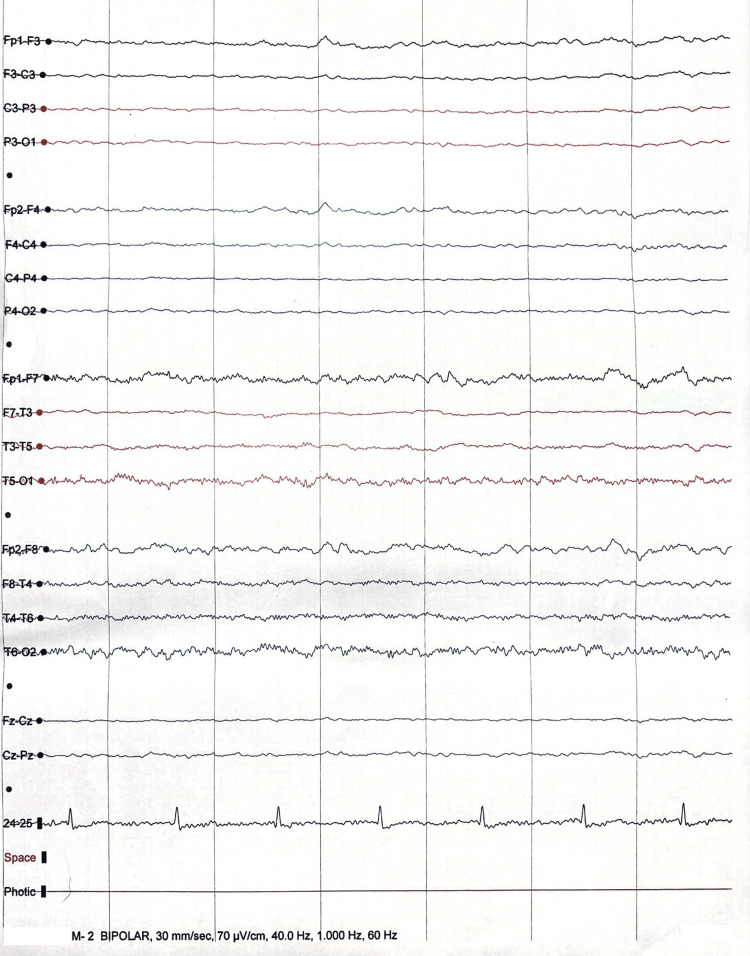
EEG negative for an epileptogenic source, but showed slowness of tracing, suggesting neurodegeneration

At the current presentation, the patient complained of chest discomfort, associated with agitation, which required the administration of haloperidol and lorazepam. She was admitted with a diagnosis of atrial fibrillation with fast ventricular response, which resolved with diltiazem. Due to the persistence of hypoactive delirium, a magnetic resonance imaging (MRI) of the brain was performed, which revealed diffuse brain involution and advanced hippocampal atrophy, typical of Alzheimer’s disease. An electroencephalogram (EEG) was negative for an epileptogenic source, but showed slowness of tracing, suggesting neurodegeneration (Figure 1).

These findings, along with a history of sudden behavioral changes, rhabdomyolysis, and a known increased risk of seizures in patients with Alzheimer’s disease, led us to suspect partial complex seizures. Consequently, intravenous LEV was started at a dose of 500 mg every 12 hours. On the fourth day of therapy, the dose was increased to 1,000 mg. LEV serum level was on target. The dose was titrated due to concern for seizure activity and was tolerated without immediate neurologic adverse effects. 

Her baseline hemoglobin was 13.0 g/L. On the second day of LEV, the hemoglobin decreased to 10.7 g/L, and on the eighth day, it further decreased to 8.4 g/L. These changes are illustrated in Figure 1. The patient remained hemodynamically stable, did not require blood transfusions, and no bleeding was found.

**Figure 2:**
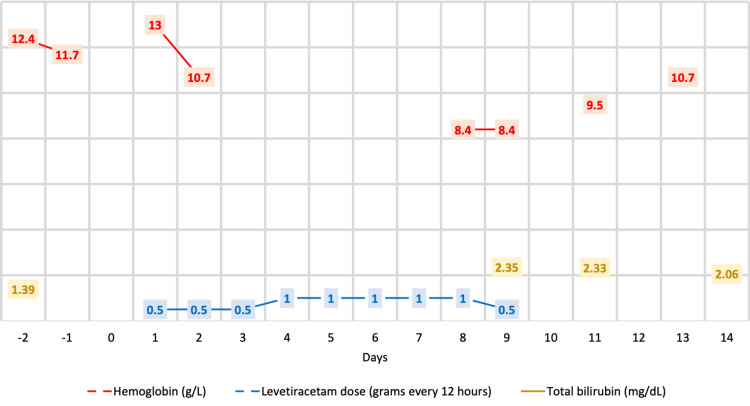
Timeline of hemoglobin and total bilirubin fluctuations in response to treatment with levetiracetam

The physical exam was remarkable for jaundice and no splenomegaly or hepatomegaly. Suspecting drug-induced anemia, LEV was discontinued after nine days of treatment. Two days after discontinuation, the hemoglobin increased to 9.5 g/L, and another two days later, it increased to 10.7 g/L. The white blood cells and platelet count were stable during these hemoglobin fluctuations.

The peripheral smear showed ovalocytes and hypochromia. Iron studies ruled out iron deficiency anemia, and inflammatory markers were normal. Vitamin B12, folic acid, and methylmalonic acid were normal as well. Other systemic causes of anemia were ruled out by the following normal laboratory results: thyroid-stimulating hormone, HIV 1/2 antigen/antibody, and rapid plasma reagin. The antinuclear antibodies screening test was positive, showing a nuclear, homogenous pattern. Thinking that an autoimmune disease could be related to this anemia and because of her history of Sjögren syndrome, the following tests were collected and returned within normal limits: complements C3 and C4, rheumatoid factor, and Sjögren antibodies anti-Ro and anti-La. The acute decline in hemoglobin was associated with an increase in reticulocyte percent. Hemolysis was suspected due to high bilirubin levels (total and indirect), elevated lactate dehydrogenase (LDH), and low haptoglobin. The ceruloplasmin level was normal, while glucose-6-phosphate dehydrogenase (G6PD) was marginally elevated. Direct and indirect Coombs tests were negative. Serum sodium and potassium levels were within normal limits throughout hospitalization. Laboratory test results are shown in Table 1. This clinical scenario was suggestive of LEV-induced hemolytic anemia.

**Table 1:** Anemia-related laboratory test results

Laboratory	Result	Reference Range	Unit
Creatinine phosphokinase (CPK)	2,928	30-135	U/L
Levetiracetam serum level	24.7	12.0-46.0	mcg/mL
Mean corpuscular volume (MCV)	87.1-91.0	80.0-99.0	fL
Red cell distribution width (RDW)	13.1-14.3	11.5-14.5	%
Hypochromia on peripheral smear	2+		
Ovalocytes on peripheral smear	1+		
Serum iron	34	37-170	mcg/dL
Ferritin	115.0	11.10-264.0	ng/mL
Total iron-binding capacity (TIBC)	228	265-497	mcg/dL
Transferrin	148	206-381	mg/dL
Transferrin saturation (TSTAT)	15	11-56	%
Erythrocyte sedimentation rate (ESR)	44	44	mm/hr
C reactive protein (CRP)	Non-reactive	Non-reactive	
Vitamin B12	338	239-931	pg/mL
Folic acid	7.54	2.76-20.0	ng/mL
Methylmalonic acid (MMA)	108	87-318	nmol/L
Prothrombin time (PT)	11.6	9.5-12.1	secs
International normalized ratio (INR)	1.0		
Partial thromboplastin time (PTT)	27.9	25.0-39.0	secs
Fibrinogen antigen	292	<350	mg/dL
D-dimer	1.24	0.00-0.49	mcg/mL FEU
Thyroid stimulating hormone (TSH)	1.030	0.465-4.680	mIU/mL
HIV ½ antigen/antibody	Non-reactive	Non-reactive	
Rapid plasma regain (RPR)	Non-reactive	Non-reactive	
Antinuclear antibodies screen (ANA)	Positive	Negative	
Antinuclear antibodies titers	1:320	>1:80	
Antinuclear antibodies pattern	Nuclear, homogenous		
Complement C3	98.0	88-165	mg/dL
Complement C4	22.30	14-44	mg/dL
Rheumatoid factor (RF)	Non-reactive	Non-reactive	
Anti-Ro (SS-A)	<0.3	0	u/mL
Anti-La (SS-B)	<0.3	0	u/mL
Reticulocytes	4.1	0.5-2.5	%
Total bilirubin	2.35	0.20-1.30	mg/dL
Indirect bilirubin	2.44	0.00-1.10	mg/dL
Direct bilirubin	<0.0	0.00-0.30	mg/dL
Lactate dehydrogenase (LDH)	471	120-246	U/L
Haptoglobin	35	43-212	mg/dL
Ceruloplasmin	25	18-53	mg/dL
Glucose-6-phosphate dehydrogenase (G6PD)	21.1	7.0-20.5	U/g Hgb
Direct Coombs test	Negative	Negative	
Indirect Coombs test	Negative	Negative	

## Discussion

In our patient, evaluation revealed reticulocytosis, not occurring in the setting of bleeding or repletion of iron, vitamin B12, folate, or copper. This, in addition to jaundice with indirect hyperbilirubinemia, elevated LDH, and decreased haptoglobin, was suggestive of hemolysis. Wilson's disease, an uncommon cause of hemolysis, was ruled out by a normal ceruloplasmin level. G6PD level was mildly elevated, which does not exclude its deficiency as the cause of hemolysis, but there have been no reports of hemolytic anemia due to G6PD deficiency in those taking LEV [4]. There were no schistocytes on the blood smear to suggest mechanical destruction. Both direct and indirect Coombs tests were negative, suggesting non-immune etiology [5]. The administration and discontinuation of LEV coincided with the acute decline and recovery of hemoglobin, indicative of LEV-induced hemolytic anemia [6-8]. Consistent with this was the normal LEV serum level.

In most cases, it is impossible to predict if a patient is going to develop an adverse drug reaction; therefore, it is good practice to be as certain as possible of the need for a specific therapy. LEV was selected for its effectiveness in treating partial seizures and its favorable safety profile in the geriatric population. Pancytopenia has been reported with the use of LEV [9], but not isolated anemia. The strengths of this case report are the extensive laboratory workup available and the evident time relationship between the offending agent administration and the drop in hemoglobin. Lowering the initial dose and slow titration of this preferred anticonvulsive drug lessened the severity of this rare but potentially serious adverse reaction.

## Conclusions

This case highlights a rare but serious adverse reaction to LEV in a 77-year-old woman with suspected Alzheimer's disease, who developed hemolytic anemia following its administration. Despite LEV’s generally favorable safety profile in the elderly, clinicians should be aware of the potential for hematologic complications. The temporal association between LEV initiation and the hemoglobin decline, along with recovery post discontinuation and supporting laboratory evidence of hemolysis, strongly suggests LEV-induced hemolytic anemia.

This case underscores the importance of closely monitoring hematologic parameters in older adults started on anticonvulsants, particularly when underlying neurodegenerative and autoimmune conditions are present. In similar clinical scenarios, initiating treatment at lower doses with gradual titration may reduce the risk of severe adverse effects.
